# The prognostic value of early measures of the ventilatory ratio in the ARDS ROSE trial

**DOI:** 10.1186/s13054-022-04179-7

**Published:** 2022-09-29

**Authors:** Ana Carolina Costa Monteiro, Sitaram Vangala, Katherine D. Wick, Kevin L. Delucchi, Emily R. Siegel, B. Taylor Thompson, Kathleen D. Liu, Anil Sapru, Pratik Sinha, Michael A. Matthay, NHLBI PETAL Network

**Affiliations:** 1 Department of Medicine, UCLA, Los Angeles, CA USA; 2 Department of Medicine, UCSF, San Francisco, CA USA; 3 Department of Psychiatry, UCSF, San Francisco, CA USA; 4 Department of Medicine, Massachusetts General Hospital, Boston, MA USA; 5 Department of Pediatrics, UCLA, Los Angeles, CA USA; 6 Department of Anesthesiology, Washington University, St. Louis, MO USA

**Keywords:** Ventilatory ratio, VR, ARDS, Neuromuscular blockade, APACHE-III, ROSE trial

## Abstract

**Background:**

The ventilatory ratio (VR, [minute ventilation × PaCO_2_]/[predicted body weight × 100 × 37.5]) is associated with mortality in ARDS. The aims of this study were to test whether baseline disease severity or neuromuscular blockade (NMB) modified the relationship between VR and mortality.

**Methods:**

This was a post hoc analysis of the PETAL-ROSE trial, which randomized moderate-to-severe ARDS patients to NMB or control. Survival among patients with different VR trajectories or VR cutoff above and below the median was assessed by Kaplan–Meier analysis. The relationships between single-day or 48-h VR trajectories with 28- or 90-day mortality were tested by logistic regression. Randomization allocation to NMB and markers of disease severity were tested as confounders by multivariable regression and interaction term analyses.

**Results:**

Patients with worsening VR trajectories had significantly lower survival compared to those with improving VR (*n* = 602, *p* < 0.05). Patients with VR > 2 (median) at day 1 had a significantly lower 90-day survival compared to patients with VR ≤ 2 (HR 1.36, 95% CI 1.10–1.69). VR at day 1 was significantly associated with 28-day mortality (OR = 1.40, 95% CI 1.15–1.72). There was no interaction between NMB and VR for 28-day mortality. APACHE-III had a significant interaction with VR at baseline for the outcome of 28-day mortality, such that the relationship between VR and mortality was stronger among patients with lower APACHE-III. There was a significant association between rising VR trajectory and mortality that was independent of NMB, baseline PaO_2_/FiO_2_ ratio and generalized markers of disease severity (Adjusted OR 1.81, 95% CI 1.28–2.84 for 28-day and OR 2.07 95% CI 1.41–3.10 for 90-day mortality). APACHE-III and NMB were not effect modifiers in the relationship between VR trajectory and mortality.

**Conclusions:**

Elevated baseline and day 1 VR were associated with higher 28-day mortality. The relationship between baseline VR and mortality was stronger among patients with lower APACHE-III. APACHE-III was not an effect modifier for the relationship between VR trajectory and mortality, so that the VR trajectory may be optimally suited for prognostication and predictive enrichment. VR was not different between patients randomized to NMB or control, indicating that VR can be utilized without correcting for NMB.

**Supplementary Information:**

The online version contains supplementary material available at 10.1186/s13054-022-04179-7.

## Introduction

Multiple pathways have been implicated in acute respiratory distress syndrome (ARDS) [1–5], including endothelial and epithelial dysfunction, immune cell recruitment and increased coagulation [6–12]. However, clinical trials have not identified effective pharmaceutical therapies, in part because the heterogeneity of ARDS represents a barrier to the identification of therapeutic targets. This has prompted the investigation of ARDS sub-phenotypes and endotypes with distinct features that may have differential responses to therapy [6, 13–20].

An understudied endotype in ARDS is defined by elevated physiologic dead space, defined as the portion of tidal volume that does not take part in gas exchange [21, 22]. Physiologic dead space is calculated by the dead space fraction, which is the ratio of dead space to tidal volume (Vd/Vt) and is a result of relative hypoperfusion of alveolar units caused by shock, overventilation of non-injured lung, compromise of the vascular bed via endothelial damage, or thrombosis [21, 23]. Indeed, elevated Vd/Vt on day 1 after intubation is a strong predictor of mortality in ARDS, making it a valuable prognostic tool that outperforms markers such as the Simplified Acute Physiology Score (SAPS) II [24], oxygenation index and driving pressure in predicting mortality [3, 22]. However, assessment of the dead space fraction requires specialized equipment and is seldom used in clinical practice [23]. Thus, a reliable and easily obtained surrogate that estimates dead space would help us prognosticate clinical outcomes in ARDS and even enable predictive enrichment for clinical studies.

The ventilatory ratio (VR), calculated as [minute ventilation (ml/min) × PaCO_2_ (mm Hg)]/(predicted body weight (kg) × 100 × 37.5), is a surrogate for dead space that is easily obtained at the bedside with an arterial blood gas and assessment of the minute ventilation [25–30]. VR measurements early after intubation have been shown to be associated with mortality in a large clinical trial and observational cohorts of ARDS [29], and in a more recent study, the trajectories of VR in intubated patients with COVID-19 were a strong predictor of mortality [31]. VR is not an exact correlate of pulmonary dead space because it is also influenced by CO_2_ production [25–29]. Conditions and interventions that might modify the relationship between VR and clinical outcomes have not been previously studied. In theory, VR might perform differently among patients receiving neuromuscular blockade (NMB) compared to those not receiving NMB, as skeletal muscle metabolism may lead to increased CO_2_ production in the latter group [32]. To that end, the validity of VR as a prognostic marker in patients treated with NMB is untested, as prior studies evaluating VR did not adjust for its use [29, 31]. In addition, ARDS in its severest forms is commonly complicated by multiorgan failure, and preexisting organ failure substantially contributes to mortality [12, 14, 33, 34]. As such, it is not known to what extent the relationship between VR and adverse outcomes differs by baseline severity of illness.

The primary objective was to investigate whether neuromuscular blockade modified the relationship between VR and mortality in the Reevaluation of Systemic Early Neuromuscular Blockade (ROSE) trial [35]. We tested for the first time whether elimination of skeletal muscle activity by neuromuscular blockade is a significant effect modifier in the relationship between VR and mortality in ventilated patients with moderate-to-severe ARDS by utilizing mixed effects modeling and multivariable logistic regression with interaction term analysis. In addition, because the ROSE cohort had high mortality and APACHE-III scores [34] compared to other ARDS and PETAL cohorts, we analyzed the data from this cohort to test whether baseline severity of illness at enrollment is a separate effect modifier to the prognostic value of VR, as baseline organ dysfunction could result in other, extra-pulmonary causes of mortality.

## Methods

### Study population

We studied participants enrolled in the ROSE trial, an unblinded multicenter randomized clinical trial run by the NHLBI Prevention and Early Treatment of Acute Lung Injury (PETAL) network that enrolled 1006 patients randomized 1:1 to NMB or usual care with light sedation targets [35]. Intubated patients were eligible for enrollment if they developed moderate-to-severe ARDS (P/F < 150) within 48 h of mechanical ventilation using PEEP of 8 cm H_2_O or greater. Exclusion criteria included pregnancy, receipt of extracorporeal membrane oxygenation (ECMO), chronic hypercapnia (PaCO_2_ > 60), chronic mechanical ventilation and bone marrow transplant within 1 year. Other study conditions were previously described [35]. The study was stopped early for futility. We excluded 2 patients in this sub-study due to issues related to data input error (both due to dates of discharge/death preceding enrollment), resulting in 1004 subjects. A total of 874 patients had VR recorded at baseline, and thus, for analysis of VR, the total n at baseline was 874.

### Selected outcomes and variables

We selected 28-day mortality as the primary outcome for this study. 90-day mortality was the secondary outcome. VR was calculated as [minute ventilation (ml/min) × PaCO_2_ (mm Hg)]/(predicted body weight (kg) × 100 × 37.5] and was collected at baseline (immediately prior to randomization), days 1–4 and day 7. The trajectory of VR over the first 2 study days was calculated as the average of the slopes of VR from baseline to day 1 and from baseline to day 2. Patients with any missing VR measurements in the first two days were excluded from trajectory analysis. Baseline and day 1 VR were categorized into a binary variable above or below the median value of 2 and used as a separate variable. VR trajectories were categorized into tertiles which were labeled as “unchanged”, “improving” or “worsening” VR trajectories. Adjustment variables were selected a priori according to clinical relevance and included the PaO_2_/FiO_2_ ratio, randomization to NMB, vasopressor use (binary) and APACHE-III score, which were collected at baseline. For these adjustment variables, other timepoints beyond baseline were not included due to complexity of their interactions and co-linearity with the variables of interest in the setting of disease progression. Measurements of PaO_2_/FiO_2_ were adjusted to atmospheric pressure for high altitude sites (> 1000 m). Predicted body weight was calculated from patient’s height, measured in supine position with legs straight. The highest and lowest 5 values from the entire study population for VR on each day were evaluated for physiologic feasibility. If the data point was outside of feasible physiologic range, as suggested by prior studies [25–29, 31] it was assumed to be an input error and excluded before further analysis was performed. As a result, a total of 11 VR measurements were removed from all timepoints, n, as indicated in results section. Missing data were not imputed.

### Power calculation

Given a final cohort of 1004 patients with a mortality rate of 35.7% (*N* = 328) at 28 days, we estimated 80% power to detect an interaction odds ratio (OR) between VR and NMB of 2.08, assuming a median split of VR, null main effects, a logistic regression and a two-sided alpha of 0.05.

### Statistical methods

Continuous variables are presented as median (IQR). Categorical variables are presented as n (%). Student’s t test was used to test for a statistical difference between two continuous variables that are approximated by a normal distribution. The Wilcoxon rank sum test was used to test for a statistical difference between two continuous variables with a skewed distribution. A chi-squared test for proportions was used to test statistical differences between frequencies of categorical variables.

Linear mixed effects modeling, with repeated measures of VR as the dependent variable and NMB and as the independent variable, tested as covariate or interaction variable along with time, was used to test whether NMB affected repeated measures of VR.

The association between VR and mortality was tested in several ways. Univariable and multivariable logistic regression was used to estimate and test the relationship between continuous and categorical baseline or day 1 VR and mortality or 2-day VR trajectory and mortality. Kaplan–Meier survival curves were plotted, and a log-rank test was used to compare survival curves. A Cox proportional regression model was used to calculate the hazard ratio (HR) for survival between the different categorical groups.

The interaction between NMB treatment group and VR; APACHE-III score and VR; baseline PaO_2_/FiO_2_ and VR; or baseline shock and VR were tested in separate logistic regression models for 28-day and 90-day mortality. These interaction analyses were performed for baseline VR, day 1 VR and VR trajectory.

A two-sided p value of 0.05 was considered statistically significant for all analyses. We did not adjust for multiple comparisons. We performed all analyses on R Studio Version 1.2.1335 software.

## Results

### Distribution of the ventilatory ratio among study participants

**Fig. 1:**
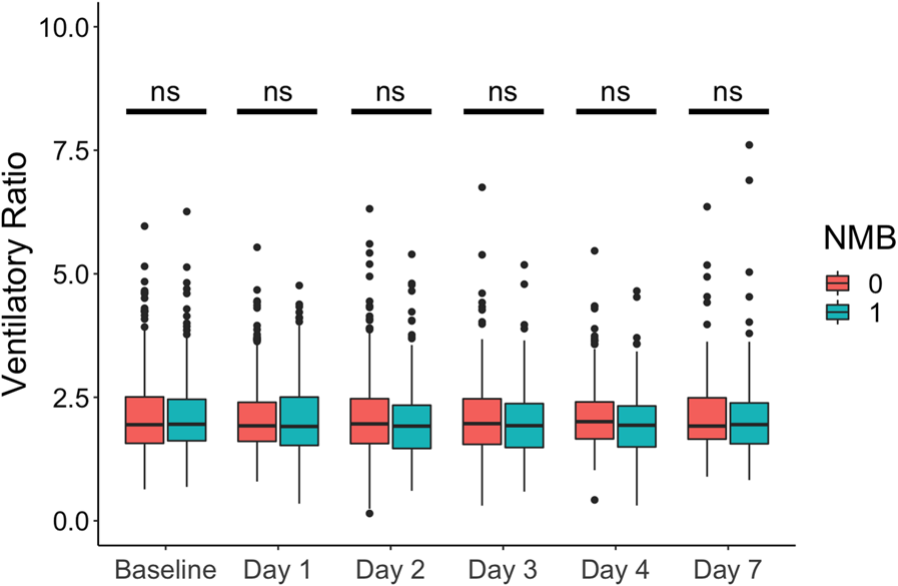
Daily distribution of ventilatory ratio (VR) categorized by randomization control (0) or NMB (1). N for subgroups after censoring (control and NMB groups, respectively), 874 at baseline (434 and 440), 808 at day 1 (386 and 422), 693 at day 2 (325 and 368), 561 at day 3 (249 and 312), 487 at day 4 (220 and 267) and 307 at day 7 (144 and 163), *p* values per Wilcoxon rank sum test

The baseline characteristics of the study population have been previously published. Briefly, there were no differences in age, sex and race between patients who received NMB blockade and those who did not [35]. The median VR was 1.95 (range 0.64–6.23) at baseline and 1.92 (range 0.35–5.54) on day 1, median 1.94 (0.15–6.32) for day 2, median 1.93 (0.31–6.75) for day 3, median 1.97 (0.31–5.47) for day 4 and median 1.94 (0.83–7.61) on day 7. There was no significant difference in VR between randomization groups at any recorded timepoint (Fig. 1).

The median baseline VR of approximately 2 is similar to what was observed in prior studies and has been previously demonstrated to have prognostic significance [29]. We therefore categorized patients based on whether they had baseline VR above or below 2. The baseline characteristics of age and race were not significantly different between the two VR categories (Table 1). The higher VR category had significantly more females than the lower VR category (VR > 2 category was 54% female, and the VR ≤ 2 category was 36% female, *p* < 0.001, Table 1). There was no difference in randomization to the NMB group between VR categories at baseline.

**Table 1:** Baseline characteristics categorized by baseline VR

	VR = < 2 (*N* = 468)	VR > 2 (*N* = 406)	*P* value
Age (*n* = 692)			
Mean (SD)	56.1 (15.9)	56.7 (15.2)	NS
Median [Min, Max]	58.0 [18.0, 91.0]	58.0 [18.0, 94.0]	
Missing	90 (19.2%)	92 (22.7%)	
Race (*n* = 874)			
Non-White	140 (29.9%)	125 (30.8%)	NS
White	328 (70.1%)	281 (69.2%)	
Sex (*n* = 874)			
Female	170 (36.3%)	221 (54.4%)	< 0.001
Male	298 (63.7%)	185 (45.6%)	
Randomization (*n* = 874)			
NMB (intervention)	232 (49.6%)	208 (51.2%)	NS
Usual care (control)	236 (50.4%)	198 (48.8%)	

Total *n* was dependent on data availability for each variable (total patients with baseline VR, *n* = 874)

### Higher VR is associated with mortality

**Fig. 2:**
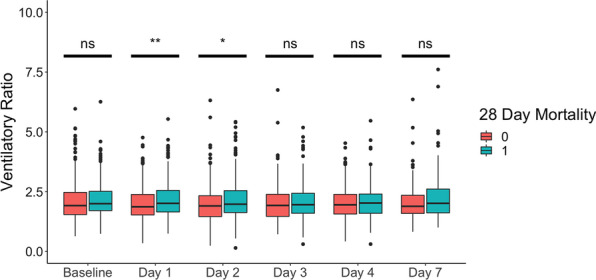
Daily distribution of ventilatory ratio (VR) categorized by 28-day survivor (0) or non-survivor (1). N for subgroups (survivor and non-survivor groups, respectively), 874 at baseline (5658 and 309), 811 at day 1 (532 and 276), 693 at day 2 (459 and 234), 561 at day 3 (374 and 187), 487 at day 4 (325 and 162) and 307 at day 7 (203 and 104). *p* values, **p < 0.01, *p < 0.05, per Wilcoxon rank sum test

Compared to survivors at 28 days, non-survivors had higher VR at baseline (2.13 vs. 2.06 for survivors, *p* < 0.1), at day 1 (2.17 vs. 2.00 for survivors, *p* < 0.01), and day 2 (2.17 vs. 1.98 for survivors, *p* < 0.01). There were no significant differences in VR observed at later timepoints beyond day 2 (Fig. 2). Compared to survivors at 90 days, non-survivors had no statistically significant difference in VR at baseline (2.11 vs. 2.01 for survivors, NS), but had a significantly higher VR at day 1 (2.15 vs. 1.99 for survivors, *p* < 0.01), and day 2 (2.13 vs. 1.98 for non-survivors, *p* = 0.05).

Since early data points (before day 2) would be most useful in identifying high-risk patients in a clinical setting, we next utilized logistic regression to test whether there was a continuous relationship between VR at baseline and VR on day 1 and mortality. Baseline VR was not significantly associated with 28-day mortality (OR = 1.14, 95% CI 0.94–1.37); however, each one-unit increase in VR on day 1 was significantly associated with 28-day mortality by univariable logistic regression (OR = 1.40, 95% CI 1.15–1.72).

### The association between VR and mortality is stronger among patients with lower APACHE-III

Given concerns for confounding to the above-observed relationships, we queried whether measures of baseline disease severity including those of pulmonary-specific disease severity selected a priori modified the relationship between VR and mortality. We tested whether APACHE-III and vasopressor use (measures of general disease severity) or PaO_2_/FiO_2_ ratio (a measure of pulmonary disease severity) at study enrollment were effect modifiers of the relationship between VR and mortality by testing separate interaction terms in individual models. There was a significant interaction between APACHE-III score and baseline VR for the outcome of 28-day mortality (Additional file 1: Table S1), with a weaker association between baseline VR and mortality as APACHE-III score increased. There were no significant interactions between other markers of disease severity (vasopressor use and P/F ratio, data not shown) and VR with the outcome of 28-day mortality. We incorporated the interaction term between APACHE-III and baseline VR in all multivariable logistic regression analyses at baseline. Because sex differed significantly by VR category (Table 1), we also tested the interaction between baseline or day 1 VR and sex for both 28-day mortality and found no significant interaction (*p* = NS).

### Higher VR is associated with mortality after adjusting for baseline markers of disease severity

We next utilized multivariable analysis to create our unified model. In separate multivariable analyses, baseline and day 1 VR were independently associated with 28-day mortality after adjusting for sex, P/F ratio, vasopressor use, APACHE-III score and the interaction term between VR and APACHE-III when applicable (Tables 2, 3). Similar trends in association were observed for the described predictor variables and 90-day mortality.

**Table 2:** Multivariable logistic regression, baseline VR (*n* = 789)

	28-day mortality			90-day mortality		
	OR	CI	*p*	OR	CI	*p*
VR	2.60	1.15–5.85	< 0.05	2.35	1.06–5.19	< 0.05
APACHE-III	1.04	1.02–1.06	< 0.001	1.04	1.03–1.06	< 0.001
Vasopressor use	1.48	1.05–2.09	< 0.05	1.39	1.00–1.93	< 0.05
PaO_2_/FiO_2_	1.00	0.99–1.00	NS	1.00	1.00–1.00	NS
VR × APACHE-III	0.99	0.98–1.00	< 0.05	0.99	0.99–1.00	< 0.05
Sex (male)	1.13	0.82–1.56	NS	1.11	0.82–1.52	NS

**Table 3:** Multivariable logistic regression, day 1 VR (*n* = 704)

	28-day mortality			90-day mortality		
	OR	CI	*p*	OR	CI	*p*
VR	1.24	1.03–1.51	< 0.05	1.37	1.09–1.73	< 0.01
APACHE-III	1.02	1.01–1.03	< 0.001	1.02	1.02–1.03	< 0.001
Vasopressor use	1.61	1.13–2.32	< 0.01	1.45	1.03–2.05	< 0.05
PaO_2_/FiO_2_	1.00	0.99–1.00	NS	1.00	1.00–1.01	NS
Sex (male)	1.19	0.86–1.67	NS	1.08	0.78–1.50	NS

**Fig. 3:**
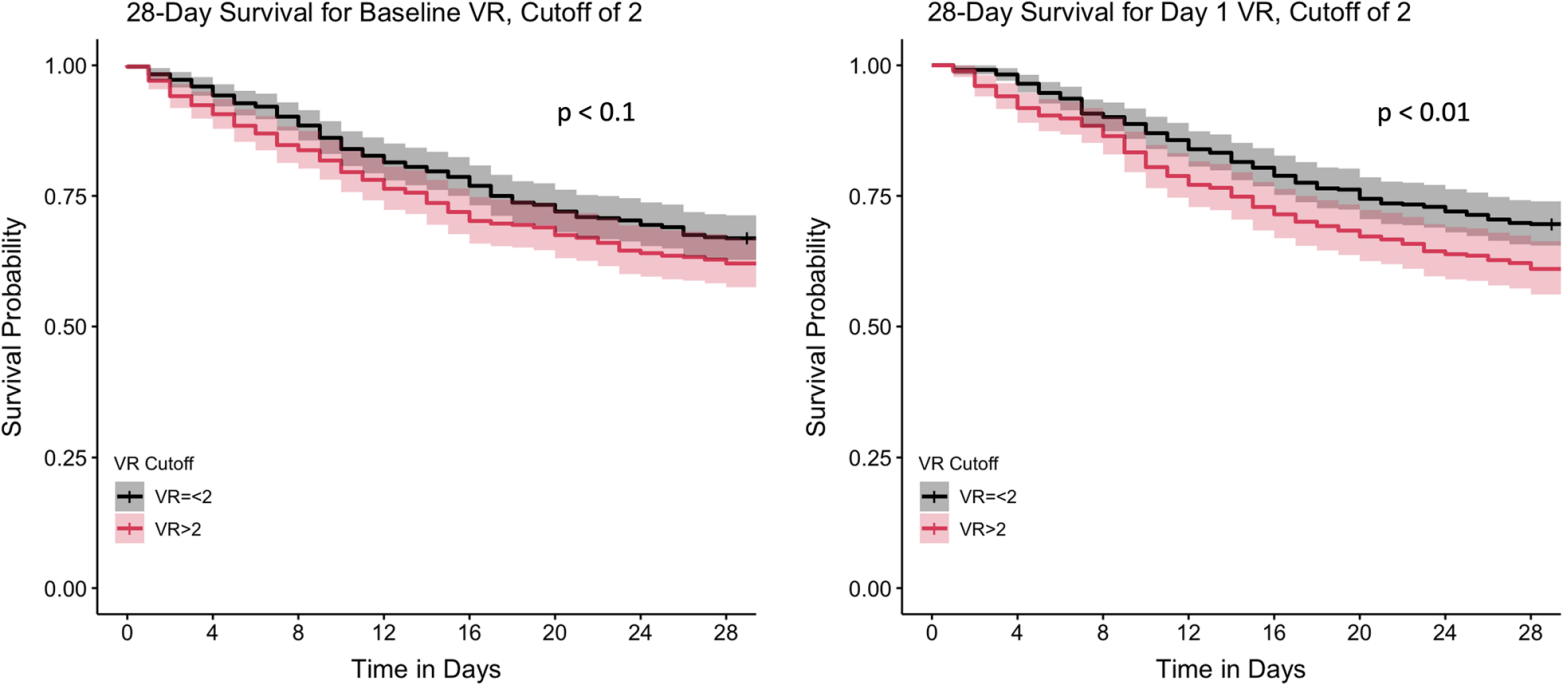
Kaplan–Meier survival curves for patients with VR equal to or below 2 versus those with VR above 2. The outcome measured was 28-day survival. Left, 28-day survival for baseline VR values (*n* = 874, *p* < 0.1 (NS), log-rank test), Right, 28-day survival for day 1 VR. Values (*n* = 808, *p* < 0.01, log-rank test)

We next asked whether VR measured at baseline or day 1 had a longitudinal effect on survival by use of unadjusted Cox proportional hazard analysis for both 28- and 90-day mortality. We compared survival among patients with a VR > 2 to those with a VR ≤ 2. Compared to the group with VR > 2, unadjusted baseline VR ≤ 2 had no statistically different probability of survival at both 28 (HR 1.21, 95% CI 0.97–1.51, *n* = 874, Fig. 3) and 90 days (HR 1.21, 95% CI 0.94–1.42, *n* = 874, Additional file 1: Fig. S1). Patients with day 1 VR of 2 or below had a statistically significantly higher survival rate at 28 days (HR 1.38, 95% CI 1.09–1.75, *n* = 808, Fig. 3) and 90 days (HR 1.36, 95% CI 1.10–1.69, *n* = 808, Additional file 1: Fig. S1) compared to patients with day 1 VR above 2. So, for unadjusted survival analysis, lower day 1 VR was significantly associated with higher probability of survival at both 28 and 90 days.

### NMB does not modify VR or the relationship between VR and mortality

Linear mixed effect modeling was utilized to test whether randomization to NMB or control (minimal sedation) affected repeated measures of VR on all recorded days (baseline to day 4 and day 7). NMB did not significantly modify the rate of change of VR (est. − 0.02, 95% CI − 0.11 to 0.06) nor the level of VR (est. − 0.04, 95% CI − 0.12 to 0.04). In addition to the observation that VR was not significantly different between randomization groups at any specific timepoint (Fig. 1), these results suggest that randomization to NMB imparts no effect modification on VR in this cohort.

Next, we asked whether randomization of NMB affected the relationship between VR and mortality. Given that randomization occurred at the baseline timepoint, we tested whether randomization to NMB affected the relationship between day 1 VR and mortality by utilizing interaction term analysis. There was no significant interaction between NMB therapy and day 1 VR in a logistic regression model of 28-day mortality or 90-day mortality (Table 4). Adding NMB as a covariate in a multivariable analysis model with day 1 VR as a predictor variable and 28-day or 90-day mortality as the outcome variables did not reveal a significant contribution of NMB to mortality in the model, nor was NMB a significant confounder in the relationship between VR and mortality (Table 5). We incorporated baseline markers of disease severity (PaO_2_/FiO_2_ ratio, APACHE-III and vasopressor use at enrollment) as covariables selected a priori.

**Table 4:** Interaction term analysis for the effect of neuromuscular blockade on the association of day 1 VR and mortality (*n* = 808)

	28-day mortality			90-day mortality		
	OR	CI	*p*	OR	CI	*p*
VR	1.57	1.16–2.13	< 0.01	1.50	1.12–2.04	< 0.01
NMB	1.39	0.56–3.47	NS	1.32	0.55–3.20	NS
VR × NMB	0.82	0.54–1.23	NS	0.85	0.57–1.27	NS

**Table 5:** Multivariable regression of day 1 VR and baseline severity indices for mortality outcomes after adding randomization to NMB to the model (*n* = 704)

	28-day mortality			90-day mortality		
	OR	CI	*p*	OR	CI	*p*
VR	1.35	1.07–1.70	< 0.05	1.36	1.08–1.71	< 0.01
APACHE-III	1.02	1.01–1.03	< 0.001	1.02	1.02–1.03	< 0.001
Vasopressor use	1.56	1.09–2.24	< 0.05	1.45	1.03–2.05	< 0.05
PaO_2_/FiO_2_	1.00	0.99–1.00	NS	1.00	1.00–1.01	NS
NMB	0.97	0.70–1.35	NS	1.02	0.74–1.41	NS

### Early changes in VR are associated with 28- and 90-day mortality

In addition, we tested whether the early trajectory of VR within the first 2 days was associated with mortality. Out of 874 patients with VR recorded at baseline, 37 died and 235 others had missing VR values in the first 2 days, so that 602 trajectories were evaluated. The slope of the two-day VR trajectory (calculated as the average of the baseline to day 1 and baseline to day 2 slopes) was associated with 28- and 90-day mortality by univariable logistic regression (OR 1.50, 95% CI 1.06–2.14 for 28-day mortality; OR 1.61, 95% CI 1.16–2.28 for 90-day mortality) and after adjusting for randomization to NMB and baseline measures of disease severity (APACHE-III, vasopressor use and PaO_2_/FiO_2_ ratio, Table 6). Randomization to NMB or baseline markers of generalized disease severity (vasopressor use, APACHE-III score) or of pulmonary disease severity (PaO_2_/FiO_2_ ratio) showed no interaction between 2-day VR trajectories and mortality, indicating that neither randomization to NMB nor selected markers of disease severity altered the relationship between VR trajectories and mortality.

**Table 6:** Multivariate regression for mortality outcomes, 2-day VR trajectory (*n* = 547)

	28-day mortality			90-day mortality		
	OR	CI	*p*	OR	CI	*p*
VR trajectory (first 2 days)	1.89	1.28–2.84	< 0.01	2.07	1.41–3.10	< 0.001
APACHE-III	1.02	1.01–1.03	< 0.001	1.02	1.02–1.03	< 0.001
Vasopressor	1.17	0.78–1.77	NS	1.24	0.83–1.83	NS
PaO_2_/FiO_2_	1.00	0.99–1.00	NS	1.00	1.00–1.01	NS
NMB (yes)	1.01	0.69–1.46	NS	1.02	0.71–1.46	NS

**Fig. 4:**
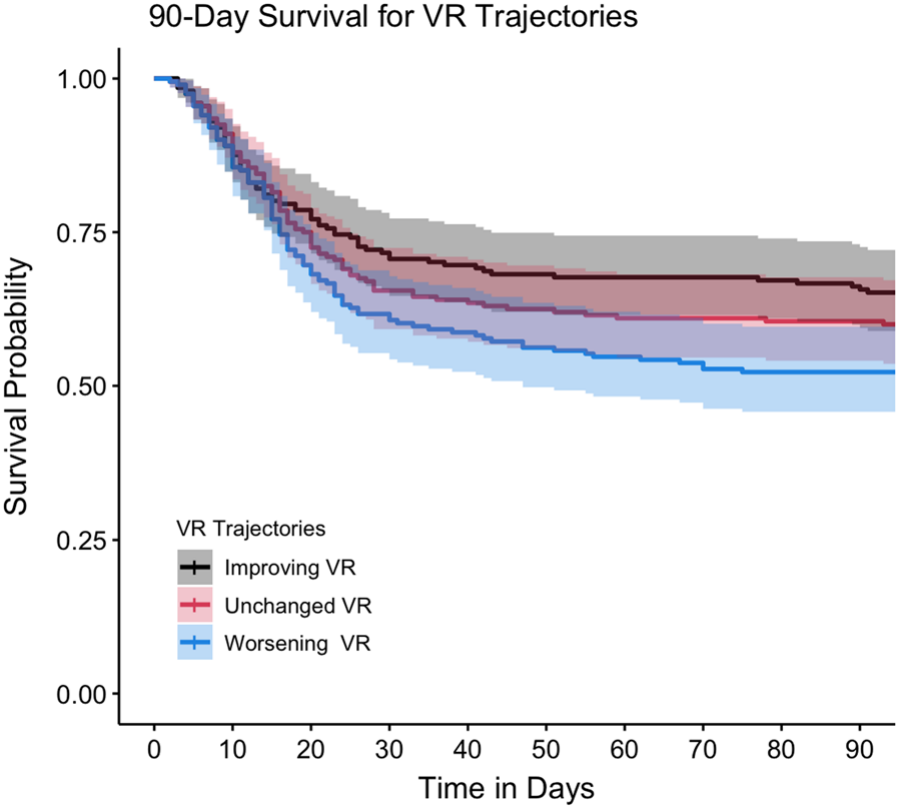
Patients with improving (downtrending) VR trajectories within the first 2 days have higher probability of 90-day survival compared to patients with worsening (up trending) VR trajectories. Kaplan–Meier survival curve with VR trajectory tertiles as categories. Improving, slope of − 2.99 to − 0.18, unchanged, slope of − 0.18 to 0.13, worsening, slope of 0.13–1.91. (*n* = 602, *p* < 0.05 for improving versus worsening groups at 90 days by Cox proportional analysis, and *p* < 0.05 comparing all three groups at 90 days, log-rank test)

Finally, patients were characterized into those with improving, unchanged or worsening VR as defined by separate tertiles of calculated trajectories. Patients with worsening VR in the first two days had a significantly greater odds of mortality compared to those with improving VR (HR 1.45, 95% CI 1.02–2.04 for 28-day mortality and HR 1.48, 95% CI 1.09–2.01 for 90-day mortality for the tertile with worsening VR compared to the tertile with improving VR tertiles by Cox proportional hazards; Table 7 and Fig. 4). There was no significant difference in mortality between the tertile with unchanged VR compared to the tertiles with either worsening or improving VRs. These data support that a worsening trajectory of VR within the first 48 h is associated with increased risk of 28- or 90-day mortality and is not subject to effect modification by baseline markers of disease severity.

**Table 7:** Comparing survival between tertiles of VR trajectory

	28-day mortality			90-day mortality		
	HR	CI	*p*	HR	CI	*p*
Unchanged (− 0.18 to 0.13)	1.25	0.88–1.79	NS	1.18	0.86–1.63	NS
Worsening (0.13–1.91)	1.45	1.02–2.04	< 0.05	1.48	1.09–2.01	< 0.05

Reference tertile for comparison is the first tertile with improving VR (downtrending, − 2.99 to − 0.18). *n* = 602

## Discussion

This study tested the prognostic value and potential effect modifiers of the ventilatory ratio in patients with moderate-to-severe ARDS who were enrolled in the ROSE trial [35]. The main findings can be summarized as follows: (1) VR was significantly higher at days 1 and 2 in non-survivors compared to survivors; (2) VR at baseline and day 1 was independently associated with 28- and 90-day mortality after adjusting for baseline APACHE-III, vasopressor requirement and PaO_2_/FiO_2_ ratio; (3) compared to an improving (downtrending) VR trajectory, a worsening (uptrending) 2-day VR trajectory was associated with higher 28- and 90-day mortality; (4) baseline APACHE-III did modify the relationship between baseline VR and 28- and 90-day mortality; and (5) NMB was not a significant effect modifier between VR and mortality.

By evaluating a large number of moderate to severe ARDS patients with high APACHE-III scores, this study provides important evidence that early VR elevation is associated with worse mortality. In addition, we tested for the first time whether NMB therapy changed VR or was an effect modifier for the relationship between VR and mortality. Since NMB blocks muscle activity, a significant contributor to CO_2_ production, we posited that NMB might alter VR and confound the relationship between VR and mortality. In theory, reducing the production of carbon dioxide by ceasing muscle contractions could modify the relationship between VR and mortality as measured CO_2_ would reflect tissue hypoperfusion and gas exchange abnormalities and not metabolic activity of muscle tissue. In addition, NMB may have affected VR by improving ventilation mechanics and optimizing dead space ventilation. Notably, NMB did not alter levels or the rate of change of VR, and there was no observed interaction between NMB and VR in models of either 28- or 90-day mortality. These findings are relevant as they support the use of VR as a prognostic method without the need for a correction term when patients are receiving NMB.

Interestingly, there was a differential effect of baseline VR on mortality by baseline APACHE-III score. Baseline VR was more strongly associated with mortality among patients with less severe baseline illness by APACHE-III. This finding indicates that VR, a moderately specific pulmonary marker, may be a less reliable prognostic indicator in cases in which baseline multiorgan failure is a major contributor to mortality. While the relationship between baseline VR and mortality differed by baseline APACHE-III, there was no such effect on the relationship between the 2-day trajectory of VR and mortality at either 28 or 90 days. Since we did not measure disease severity at later timepoints, we could not assess the interference of contemporaneous multiorgan damage to the relationship between VR and mortality. Nonetheless, an early VR trajectory may be a more generalizable prognosticator of mortality compared to single VR timepoints, as it is at least independent of baseline disease severity and thus could be a good candidate marker for selecting sicker patients for clinical trials.

The strengths of this study were the size of the cohort, the unprecedented severity of illness of the study population, and that the original study was a randomized trial that evaluated the effects of NMB on outcomes of patients with ARDS. Study limitations include: (1) This was a secondary analysis, with missing data leading to an effective study size of 874 baseline measurements, though this limitation is mitigated by the large original sample size; (2) the ROSE cohort was managed with higher PEEP than may be encountered in the clinical setting, which may have implications to the measurements of VR in the study, (3) we did not exclude patients with short stature from the analysis, which may have resulted in overestimation of VR in those patients; and (4) ventilator parameters were only available once daily, which precluded the analysis of VR trajectories by more complex statistical algorithms, as has been recently performed in a large observational cohort study [31].

Our study emphasizes the value of the ventilatory ratio as a practical prognostic marker in ARDS. For treatments that could reduce pulmonary dead space, higher VR could be used for predictive enrichment also. In summary, these findings highlight that the early trajectory of the ventilatory ratio is an important and practical prognostic method that is not substantially impacted by baseline severity of illness with the potential to be a physiologic criterion for selection of patients for clinical trials.

## Conclusions

Higher baseline, day 1 or increasing early VR trajectory is associated with increased mortality. The early VR trajectory is not modified by baseline markers of disease severity and may be a useful tool for patient selection in ARDS trials.

## Supplementary Information

**Additional file 1: Figure S1**. Kaplan–Meier survival curves for patients with VR equal to or below 2 versus those with VR above 2. The outcome measured was 90-day survival. Left, 90-day survival for baseline VR values (*n* = 874, *p* = NS, log-rank test), Right, 90-day survival for day 1 VR. Values (*n* = 808, *p* < 0.01, log-rank test). **Table S1**. Interaction term analysis, baseline VR (*n* = 790).

## Data Availability

The data for the NHLBI PETAL ROSE trial are now available through NHLBI’s repository and can be requested at https://biolincc.nhlbi.nih.gov/studies/petal_rose/.
