# Supplementary material for: The prognostic value of early measures of the ventilatory ratio in the ARDS ROSE trial
Source: Crit Care. 2022 Sep 29;26:297. doi: 10.1186/s13054-022-04179-7 (PMC9521854; doi:10.1186/s13054-022-04179-7)
Supplement: Supplementary file 1 — Additional file 1: Figure S1. Kaplan–Meier survival curves for patients with VR equal to or below 2 versus those with VR above 2. The outcome measured was 90-day survival. Left, 90-day survival for baseline VR values (n = 874, p = NS, log-rank test), Right, 90-day survival for day 1 VR. Values (n = 808, p < 0.01, log-rank test). Table S1. Interaction term analysis, baseline VR (n = 790). [file 13054_2022_4179_MOESM1_ESM.docx]

**Additional file 1: Figure S1.** **Kaplan Meier survival curves for patients with VR equal to or below 2 vs. those with VR above 2.** The outcome measured was 90-day survival. Left, 90-day survival for baseline VR values (n= 874, p=NS, log-rank test), Right, 90-day survival for Day 1 VR. Values (n=808, p<0.01, log-rank test).


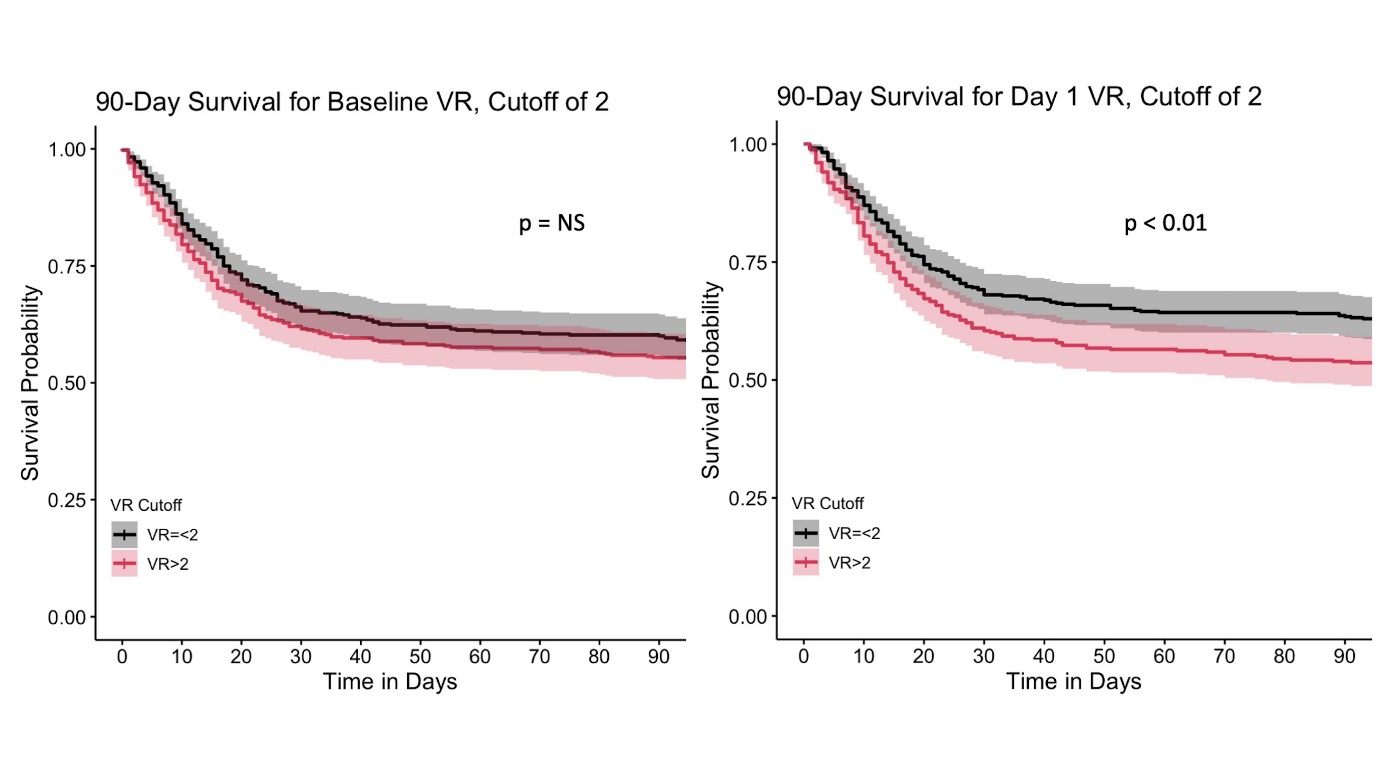


**Additional file 1: Table S1.** Interaction term analysis, baseline VR (n=790)

|  | 28-Day Mortality | | | 90-Day Mortality | | |
| --- | --- | --- | --- | --- | --- | --- |
|  | OR | CI | p | OR | CI | p |
| Baseline VR | 2.66 | 1.18 – 5.94 | <0.05 | 2.38 | 1.08 – 5.22 | <0.05 |
| APACHE-III | 1.04 | 1.03 – 1.06 | <0.001 | 1.04 | 1.03 – 1.06 | <0.001 |
| VR x APACHE-III | 0.99 | 0.98 – 1.00 | <0.05 | 0.99 | 0.98 – 1.00 | <0.05 |
